# Supplementary material for: Diagnostic test accuracy of simplified algorithms for diagnosing acute rheumatic fever: a systematic review
Source: Commun Med (Lond). 2025 Aug 12;5:348. doi: 10.1038/s43856-025-01023-1 (PMC12344290; doi:10.1038/s43856-025-01023-1)
Supplement: Supplementary file 1 — Supplementary Material [file 43856_2025_1023_MOESM1_ESM.pdf]

Supplementary Information

Supplementary Table 1. Search Strategy

Database: Embase <1974 to 2025 March 15>
1 Rheumatic Fever/ or (Rheumatic Fever* or Rheumatoid Fever*).mp. (10513)
2 exp *"Sensitivity and Specificity"/ or *Predictive Value/ or *Receiver Operating Characteristic/ or *False Negative Result/ or *False Positive Result/ or exp *Algorithm/ or exp *Laboratory Technique/ or *Clinical Chemistry/ or exp *Genetic Screening/ or exp *Immunological Procedures/ or *Critical Value/ or *Blood Culture/ or *Molecular Diagnosis/ or exp *Serology/ or *Mandatory Testing/ or *Heterozygote Detection/ or *Multiphasic Screening/ or exp *Diagnosis/ or *Differential Diagnosis/ or exp *Diagnostic Procedure/ or *Symptom Assessment/ or *Early Diagnosis/ or exp *Mass Screening/ or exp *Genetic Predisposition/ or *Genetic Susceptibility/ or *Biological Marker/ or exp *Genetic Marker/ or *Environmental Marker/ or *Tenascin/ or *Albuminoid/ or *Gamma Interferon Inducible Protein 10/ or *Antistreptolysin/ or *Antistreptolysin Test Kit/ or Diagnosis.fs. or (Sensitivity or Specificity or Diagnostic Accuracy or Diagnostic Test Accuracy or Diagnostic Performance or ROC or "Receiver Operating Characteristic" or "Receiver Operating Characteristics" or "Predictive Value" or "Predictive Values" or NPV or NPVs or PPV or PPVs or "False Positive" or "False Negative" or "True Positive" or "True Negative" or Likelihood Function* or Likelihood Estimat* or Likelihood Ratio* or "LR+" or "LR-" or "Early Detection" or "Jones Criteria" or "Jones' Criteria" or "Jones's Criteria" or Algorithm* or Simpl* or Biomarker* or Marker* or Surrogate Endpoint* or Surrogate End Point* or Bioindicator* or Biological Indicator* or Culture* or Swab* or Diagnos* or Genetic* or Gene or Genes or Heterozygote Detection* or Immunodiagnos* or Immunologic Test* or Immunological Test* or Laborator* or Lab or Labs or Mandatory Test* or Molecular Test* or Periostin or Tenascin* or Hexabrachion or Cytotactin or "J1-200-220" or Albumin* or CXCL10 or INP10 or "Interferon Induced Protein 10" or "Interferon-γ Induced Protein 10" or "Interferon-Gamma Induced Protein 10" or "Gamma Interferon Inducible Protein 10" or "IP-10" or "Mob-1 Protein" or SCYB10 or "Interferon Inducible Protein 10" or "Interferon-γ Inducible Protein 10" or "Interferon-Gamma Inducible Protein 10" or "Chemokine Ligand 10" or "Chemokine (C-X-C Motif) Ligand 10" or "Small Inducible Cytokine B10" or "GammaIP-10 Protein" or "Interferon gamma Inducible Protein of 10 kDa" or IgG3-C4 or Screen* or Serodiagnos* or Serologic Test* or Serological Test* or Symptom Assessment* or Symptom Evaluation* or Antistreptolysin or Anti-Streptolysin or "Antideoxyribonuclease B" or "Anti-Deoxyribonuclease" or Streptococcal Antigen* or Streptococcal Antibod* or Streptolysin Antibod*).mp. (21563398)
3 1 and 2 (6625)
4 (rat or rats or mouse or mice or swine or porcine or murine or sheep or lambs or pigs or piglets or rabbit or rabbits or cat or cats or dog or dogs or cattle or bovine or monkey or monkeys or trout or marmoset$1).ti. and animal experiment/ (1291773)
5 Animal experiment/ not (human experiment/ or human/) (2722080)
6 4 or 5 (2802143)
7 3 not 6 (6581)
Database: Ovid MEDLINE(R) ALL <1946 to March 15, 2025>
1 Rheumatic Fever/ or (Rheumatic Fever* or Rheumatoid Fever*).mp. (13426)
2 "Sensitivity and Specificity"/ or "Predictive Value of Tests"/ or "ROC Curve"/ or "False Negative Reactions"/ or "False Positive Reactions"/ or Likelihood Functions/ or Algorithms/ or Clinical Laboratory Techniques/ or exp Clinical Chemistry Tests/ or Genetic Testing/ or exp Immunologic Tests/ or Laboratory Critical Values/ or Blood Culture/ or Molecular Diagnostic Techniques/ or exp Serologic Tests/ or Mandatory Testing/ or Diagnostic Screening Programs/ or Genetic Carrier Screening/ or Multiphasic Screening/ or Diagnosis/ or Diagnosis, Differential/ or "Diagnostic Techniques and Procedures"/ or Symptom Assessment/ or Early Diagnosis/ or Mass Screening/ or exp Genetic Predisposition to Disease/ or Anticipation, Genetic/ or Biomarkers/ or Genetic Markers/ or Environmental Biomarkers/ or Tenascin/ or Albumins/ or Chemokine CXCL10/ or Antistreptolysin/ or (Diagnosis or Genetics).fs. or (Sensitivity or Specificity or Diagnostic Accuracy or Diagnostic Test Accuracy or Diagnostic Performance or ROC or "Receiver Operating Characteristic" or "Receiver Operating Characteristics" or "Predictive Value" or "Predictive Values" or NPV or NPVs or PPV or PPVs or "False Positive" or "False Negative" or "True Positive" or "True Negative" or Likelihood Function* or Likelihood Estimat* or Likelihood Ratio* or "LR+" or "LR-" or "Early Detection" or "Jones Criteria" or "Jones' Criteria" or "Jones's Criteria" or Algorithm* or Simpl* or Biomarker* or Marker* or Surrogate Endpoint* or Surrogate End Point* or Bioindicator* or Biological Indicator* or Culture* or Swab* or Diagnos* or Genetic* or Gene or Genes or Heterozygote Detection* or Immunodiagnos* or Immunologic

Test* or Immunological Test* or Laborator* or Lab or Labs or Mandatory Test* or Molecular Test* or Periostin or Tenascin* or Hexabrachion or Cytotactin or "J1-200-220" or Albumin* or CXCL10 or INP10 or "Interferon Induced Protein 10" or "Interferon-γ Induced Protein 10" or "Interferon-Gamma Induced Protein 10" or "Gamma Interferon Inducible Protein 10" or "IP-10" or "Mob-1 Protein" or SCYB10 or "Interferon Inducible Protein 10" or "Interferon-γ Inducible Protein 10" or "Interferon-Gamma Inducible Protein 10" or "Chemokine Ligand 10" or "Chemokine (C-X-C Motif) Ligand 10" or "Small Inducible Cytokine B10" or "GammaIP-10 Protein" or "Interferon gamma Inducible Protein of 10 kDa" or IgG3-C4 or Screen* or Serodiagnos* or Serologic Test* or Serological Test* or Symptom Assessment* or Symptom Evaluation* or Antistreptolysin or Anti-Streptolysin or "Antideoxyribonuclease B" or "Anti-Deoxyribonuclease" or Streptococcal Antigen* or Streptococcal Antibod* or Streptolysin Antibod*).mp. (16386230)
3 1 and 2 (5398)
4 exp Animals/ not Humans.sh. (5316086)
5 3 not 4 (5354)
Conference Proceedings Citation Index – Science (CPCI-S) (1990 – 2025, March 15)
(Rheumatic Fever* OR Rheumatoid Fever*) AND (Sensitivity OR Specificity OR Diagnostic Accuracy OR Diagnostic Test Accuracy OR Diagnostic Performance OR ROC OR "Receiver Operating Characteristic" OR "Receiver Operating Characteristics" OR "Predictive Value" OR "Predictive Values" OR NPV OR NPVs OR PPV OR PPVs OR "False Positive" OR "False Negative" OR "True Positive" OR "True Negative" OR Likelihood Function* OR Likelihood Estimat* OR Likelihood Ratio* OR "LR+" OR "LR-" OR "Early Detection" OR "Jones Criteria" OR "Jones' Criteria" OR "Jones's Criteria" OR Algorithm* OR Simpl* OR Biomarker* OR Marker* OR Surrogate Endpoint* OR Surrogate End Point* OR Bioindicator* OR Biological Indicator* OR Culture* OR Swab* OR Diagnos* OR Genetic* OR Gene OR Genes OR Heterozygote Detection* OR Immunodiagnos* OR Immunologic Test* OR Immunological Test* OR Laborator* OR Lab OR Labs OR Mandatory Test* OR Molecular Test* OR Periostin OR Tenascin* OR Hexabrachion OR Cytotactin OR "J1-200-220" OR Albumin* OR CXCL10 OR INP10 OR "Interferon Induced Protein 10" OR "Interferon-γ Induced Protein 10" OR "Interferon-Gamma Induced Protein 10" OR "Gamma Interferon Inducible Protein 10" OR "IP-10" OR "Mob-1 Protein" OR SCYB10 OR "Interferon Inducible Protein 10" OR "Interferon-γ Inducible Protein 10" OR "Interferon-Gamma Inducible Protein 10" OR "Chemokine Ligand 10" OR "Chemokine (C-X-C Motif) Ligand 10" OR "Small Inducible Cytokine B10" OR "GammaIP-10 Protein" OR "Interferon gamma Inducible Protein of 10 kDa" OR IgG3-C4 OR Screen* OR Serodiagnos* OR Serologic Test* OR Serological Test* OR Symptom Assessment* OR Symptom Evaluation* OR Antistreptolysin OR Anti-Streptolysin OR "Antideoxyribonuclease B" OR "Anti-Deoxyribonuclease" OR Streptococcal Antigen* OR Streptococcal Antibod* OR Streptolysin Antibod*) (Topic) 140

Supplementary Table 2. Original Jones criteria (Jones 1944)

**Major criteria**	**Minor criteria**
Carditis	Fever
Arthralgia	Abdominal pain
Chorea	Precordial pain
Subcutaneous nodules	Erythema marginatum
History of previous definite RH or RHD	Epistaxis
	Pulmonary findings
	Laboratory findings<br>Electrocardiography abnormalities<br>Microcytic anaemia<br>Elevated white blood cell count<br>Elevated erythrocyte sedimentation rate

Reference:

Jones TD. The diagnosis of rheumatic fever. JAMA 1944; 126 (8): 481–4. doi:10.1001/jama.1944.02850430015005

Supplementary Table 3. Revised Jones Criteria (Gewitz et al. 2015)

A. For all patient populations with evidence of preceding GAS infection	
Diagnosis: initial ARF	2 Major manifestations or 1 major plus 2 minor manifestations
Diagnosis: recurrent ARF	2 Major or 1 major and 2 minor or 3 minor
B. Major criteria	
Low-risk populations*	Moderate- and high-risk populations
Carditis** Clinical and/or subclinical	Carditis** Clinical and/or subclinical
Arthritis Polyarthritis only	Arthritis Monoarthritis or polyarthritis Polyarthralgia***
Chorea	Chorea
Erythema marginatum	Erythema marginatum
Subcutaneous nodules	Subcutaneous nodules
C. Minor criteria	
Low-risk populations*	Moderate- and high-risk populations
Polyarthralgia	Monoarthritis
Fever (≥38.5◦C)	Fever (≥38.5◦C)
ESR ≥60 mm in the first hour and/or CRP ≥3.0 mg/dL ****	ESR ≥30 mm/h and/or CRP ≥3.0 mg/dL ****
Prolonged PR interval, after accounting for age variability (unless carditis is a major criterion)	Prolonged PR interval, after accounting for age variability (unless carditis is a major criterion)

ARF: acute rheumatic fever; CRP: C-reactive protein; ESR: erythrocyte sedimentation rate; GAS: group A streptococcal infection.

* Low-risk populations are those with ARF incidence of ≤2 per 100,000 school-aged children or all-age RHD prevalence of ≤1 per 1000 population per year. ** Subclinical Carditis indicates echocardiographic valvulitis as defines in Table 2. *** See section on polyarthralgia, which should only be considered as a major manifestation in moderate- to high-risk populations after exclusion of other causes (Gewitz et al. 2015). As in past versions of the criteria, erythema marginatum and subcutaneous nodules are rarely “stand-alone” major criteria. Additionally, joint manifestations can only be considered in either the major or minor categories but not both in the same patient. **** CRP value must be greater than the upper limit of normal for laboratory. Also, because ESR may evolve during the course of ARF, peak ESR values should be used.

Reference: Gewitz MH, Baltimore RS, Tani LY, Sable CA, Shulman ST, Carapetis J, Remenyi B, Taubert KA, Bolger AF, Beerman L, Mayosi BM, Beaton A, Pandian NG, Kaplan EL; American Heart Association Committee on Rheumatic Fever, Endocarditis, and Kawasaki Disease of the Council on Cardiovascular Disease in the Young.Revision of the Jones

Supplementary Table 4. Doppler Findings in Rheumatic Valvulitis (Gewitz et al. 2015)

Pathological mitral regurgitation (all 4 criteria met)
Seen in at least 2 views
Jet length ≥2 cm in at least 1 view
Peak velocity >3 m/s
Pansystolic jet in at least 1 envelope
Pathological aortic regurgitation (all 4 criteria met)
Seen in at least 2 views
Jet length ≥1 cm in at least 1 view
Peak velocity >3 m/s
Pan diastolic jet in at least 1 envelope

Loading conditions should be accounted for at the time of echocardiography/Doppler assessment (see the section Differential Diagnosis of ARF for a full discussion). This table reflects an amalgam of the findings from the references listed in Table 5 (Gewitz et al. 2015) and other guideline statements (RHDAustralia 2020; Atatoa-Carr et al. 2008) and also resembles findings described in rheumatic heart disease (Reményi et al. 2012).

References:

Gewitz MH, Baltimore RS, Tani LY, Sable CA, Shulman ST, Carapetis J, Remenyi B, Taubert KA, Bolger AF, Beerman L, Mayosi BM, Beaton A, Pandian NG, Kaplan EL; American Heart Association Committee on Rheumatic Fever, Endocarditis, and Kawasaki Disease of the Council on Cardiovascular Disease in the Young.Revision of the Jones Criteria for the diagnosis of acute rheumatic fever in the era of Doppler echocardiography: a scientific statement from the American Heart Association. Circulation. 2015;131(20):1806-18. doi: 10.1161/CIR.0000000000000205.

RHDAustralia (ARF/RHD writing group). The 2020 Australian guideline for prevention, diagnosis and management of acute rheumatic fever and rheumatic heart disease (3.2 edition, March 2022); 2020

Atatoa-Carr P, Lennon D, Wilson N; New Zealand Rheumatic Fever Guidelines Writing Group. Rheumatic fever diagnosis, management, and secondary prevention: a New Zealand guideline. N Z Med J. 2008;121(1271):59-69.

Reményi B, Wilson N, Steer A, Ferreira B, Kado J, Kumar K, Lawrenson J, Maguire G, Marijon E, Mirabel M, Mocumbi AO, Mota C, Paar J, Saxena A, Scheel J, Stirling J, Viali S, Balekundri VI, Wheaton G, Zühlke L, Carapetis J. World Heart Federation criteria for echocardiographic diagnosis of rheumatic heart disease: an evidence-based guideline.Nat Rev Cardiol. 2012; 9:297–309. DOI: 10.1038/nrcardio.2012.7.

Supplementary Table 5. Reason for exclusion of studies and studies awaiting classification

Study name	Reason for exclusion
Narang *et al.* 2021[32]	Not a DTA study. Narang and colleagues derived a new stratification scheme for distinguishing ARF with carditis from latent RHD (wrong outcome). On multiple regression analysis, the following 6 variables were found to be independently predictive of ARF with carditis and used to compose a score (0-13): age (> 12 years : 0 points; 8-12: 1 point; <8 years: 2 points), NYHA class (I: 0 points; II: 1 point; III: 2 points; IV: 3 points), the severity of mitral regurgitation (nil: 0 points; mild: 1 point; moderate: 2 points; severe: 3 points), mitral leaflet nodules (absent: 0 points; present: 2 points), erythrocyte sedimentation rate (≤50: 0 points; >50: 2 points) and antistreptolysin titer (≤250: 0 points; >250: 1 point). A score of ≥5 was found to be the best threshold for diagnosing ARF with carditis (AUC ROC 0.87, sensitivity 76%, specificity 79%). Laboratory data was missing in ≥20% of patients. However, discriminative performance was assessed in the derivation cohort only. Validation in an external population is still required to confirm this score's discriminative capacity, namely for ARF vs no ARF cases in suspected individuals with fever.
Ralph *et al.* 2021[19]	Study Protocol for the START study which aims to determine the biomarker signature of ARF. The lead author clarified development of a simplified diagnostic algorithm has not been planned.<br>This study will enroll 120 ARF cases and 130 controls divided between derivation and validation cohorts. The aim is biomarker discovery, including immunophenotyping, metabolomics, blood transcriptomics, antibody analysis, among other investigations. Biomarkers identified in the study are to be used alongside the existing diagnostic criteria, as a diagnostic adjunct, rather than leading to simplification.
Fisher et al. 2022	Not a DTA study. Evaluation of a digital app to help clinicians apply the Australian diagnosis algorithm for definite, probable or possible acute rheumatic fever, based on the 2015 modified Jones criteria.
Lindhom et al. 2023	Not a DTA study. Retrospective audit of children and adolescents with ARF and RHD attending the Royal Children's and Monash Children's Hospitals in Victoria, Australia. No data available on simplified approaches for ARF diagnosis.
Machipisa et al. 2023	Study Protocol for a Cohort study for biomarker studies, including genome-wide association studies with participants from 8 African countries. Wrond population. 2548 RHD cases and 2261 controls.
Yavrum et al. 2023	Not a DTA study. Clinical audit on ARF children in pediatric cardiology clinic of Ankara City Hospital, Turkey. No data available on simplified approaches for ARF diagnosis.
Ali et al. 2024	Not a DTA study. No data presented. Conceptual algorithm for diagnosis of ARF based on arthritis, carditis and chorea, classifying patients as suspected and confirmed cases in primary care. In secondary care, further assessment with echocardiography and review of clinical history for negatives cases is recommended.
Okello et al. 2024	Awaiting Classification. No data available for DTA. Abstract only. Once published as a manuscript may need to be rescreened. Describes a cohort of 165 children, 50 with definite ARF and the remaining with other clinically overlapping conditions (no details provided). Two sites involved: central vs western recruitment site (not details provided on location). Utililizing SomaScan proteomic profiling (no details provided on version) twenty-two proteins significantly associated with ARF were identified, and 5 had very good discriminative capacity for clinical cases (not specified) when combined: central training cohort (AUC 0.97, 95% CI 0.90-0.97), and western testing cohort (AUC 0.84, 95% CI 0.71-0.84). These proteins returned to baseline on convalescent phase. No uniprot id or protein names were provided.
Panwar et al. 2025	Awaiting Classification. No data available for DTA. Abstract only. Once published as a manuscript may need to be rescreened. Describes a cohort of 200 Children with febrile illness and sore throat but lacking ARF features. Screened with echocardiogram; the abstract mentions 7 cases (3.5%) of RHD cases were discovered. However, no data on modified jones criteria and DTA with and without echocardiography is presented.

Legend: ARF – acute rheumatic fever; RHD – rheumatic heart disease; NYHA – New Yort Heart Association; AUC ROC- area under the curve receiver operating characteristic.

References:

Fisher E, James C, Mosca D, Currie BJ, Ralph AP. Evaluation of an ARF diagnosis calculator: a survey and content analysis. BMC Med Inf Decis Mak. 2022;22:77.

Lindholm DE, Whiteman IJ, Oliver J, Cheung MMH, Hope SA, Brizard CP, Horton AE, Sheridan B, Hardy M, Osowicki J, Steer AC, Engelman D. Acute rheumatic fever and rheumatic heart disease in children and adolescents in Victoria, Australia. J Paediatr Child Health. 2023;59:352-359.

Machipisa T, Chishala C, Shaboodien G, Zühlke LJ, Muhamed B, Pandie S, de Vries J, Laing N, Joachim A, Daniels R, Ntsekhe M, Hugo-Hamman CT, Gitura B, Ogendo S, Lwabi P, Okello E, Damasceno A, Novela C, Mocumbi AO, Madeira G, Musuku J, Mtaja A, ElSayed A, Alhassan HHM, Bode-Thomas F, Yilgwan C, Amusa G, Nkereuwem E, Mulder N, Ramesar R, Lesosky M,

Cordell HJ, Chong M, Keavney B, Paré G, Engel ME; RHDGen Network Consortium†. Rationale, Design, and the Baseline Characteristics of the RHDGen (The Genetics of Rheumatic Heart Disease) Network Study. Circ Genom Precis Med. 2023;16:e003641.

Narang R, Saxena A, Ramakrishnan S, Gupta SK, Juneja R, Kothari SS. An evidence-based scoring system to diagnose acute rheumatic fever with carditis in children. Int J Cardiol. 2021;333:146-51.

Ralph AP, Webb R, Moreland NJ, McGregor R, Bosco A, Broadhurst D, Lassmann T, Barnett TC, Benothman R, Yan J, Remenyi B, Bennett J, Wilson N, Mayo M, Pearson G, Kollmann T, Carapetis JR. Searching for a technology-driven acute rheumatic fever test: the START study protocol. BMJ Open. 2021;11:e053720.

Yavrum BE, Gul AEK, Azak E, Gursu HA, Cetin II. Changing face of acute rheumatic fever in childhood and our clinical results. North Clin Istanb.2023;10:237-247.

Ali SK. A new algorithm for diagnosis of acute rheumatic fever: is jones criteria reign over? Cardiol Young. 2024;34(Supplement 1):S1434.

Okello E, Beaton A, Ndagire E, Othman RB, Sable C, Barnett T, Shannon CP, Tebbutt SJ, Kollmann TR, Carapetis J. A plasma protein biomarker for acute rheumatic fever. Cardiol Young. 2024;34(Supplement 1):S1447.

Panwar RB, Agarwal DK, Panwar VR, Akhtar N, Panwar SR, Nahata P, Nanda NC, Sharma A, Panwar F, Prajapat S. Prevalence of acute rheumatic fever (sub clinical carditis) by using standard echocardiography for febrile children with sore throat presenting as rheumatic heart disease in endemic area. J Am Coll Cardiol. 2025;85(12 Supplement):2275.
