# Supplementary material for: Diagnostic test accuracy of simplified algorithms for diagnosing acute rheumatic fever: a systematic review
Source: Commun Med (Lond). 2025 Aug 12;5:348. doi: 10.1038/s43856-025-01023-1 (PMC12344290; doi:10.1038/s43856-025-01023-1)
Supplement: Supplementary file 2 — Description of Additional Supplementary Files [file 43856_2025_1023_MOESM2_ESM.pdf]

Description of Additional Supplementary Files

File name: Supplementary Data 1

Description: Quality Assessment of Diagnostic Accuracy Studies-2 (QUADAS-2) tool
